# Comparative Evaluation of Sequencing Technologies for Detecting Antimicrobial Resistance in Bloodstream Infections

**DOI:** 10.3390/antibiotics14121257

**Published:** 2025-12-12

**Authors:** Myrto Papamentzelopoulou, Georgia Vrioni, Vassiliki Pitiriga

**Affiliations:** 1 Molecular Biology Unit, 1st Department of Obstetrics and Gynecology, National and Kapodistrian University of Athens, 11527 Athens, Greece; 2 Department of Microbiology, Medical School, National and Kapodistrian University of Athens, 75 Mikras Asias Street, 11527 Athens, Greece

**Keywords:** bloodstream infections, antimicrobial resistance, next-generation sequencing, whole-genome sequencing, metagenomics, sepsis, resistome

## Abstract

Bloodstream infections (BSIs) pose a significant global health challenge, particularly due to the increasing prevalence of antimicrobial resistance (AMR). Timely and accurate identification of pathogens and resistance determinants is critical for guiding appropriate therapy and improving patient outcomes. Traditional culture-based diagnostics are limited by prolonged turnaround times and reduced sensitivity, especially in culture-negative or polymicrobial infections. This review systematically examined current and emerging sequencing technologies for AMR detection in BSIs, including whole-genome sequencing (WGS), targeted next-generation sequencing (tNGS), metagenomic next-generation sequencing (mNGS), and long-read sequencing platforms (Oxford Nanopore, PacBio). We compared their clinical performance using key metrics such as diagnostic sensitivity, turnaround time, and cost, highlighting contexts in which each technology is most effective. For example, tNGS can achieve the rapid detection of known resistance genes within 8–24 h, while WGS provides comprehensive genome-wide resistance profiling over 24–48 h. mNGS offers broader detection, including rare or unexpected pathogens, although at higher cost and longer processing times. Our analysis identifies specific strengths and limitations of each approach, supporting the use of context-specific strategies, such as combining rapid targeted sequencing for common pathogens with broader metagenomic approaches for complex cases, to improve diagnostic yield and guide antimicrobial therapy. Quantitative comparisons indicate that sequencing technologies can complement conventional methods, particularly in cases where culture-based approaches fail. In conclusion, sequencing-based diagnostics offer measurable improvements in sensitivity and speed over traditional methods for AMR detection in BSIs. Future work should focus on optimizing workflows, integrating sequencing data into clinical decision-making, and validating approaches in prospective studies.

## 1. Introduction

### 1.1. Burden of Bloodstream Infections (BSIs) and the Global Threat of AMR

Bloodstream infections (BSIs), also known as septicemia, occur when harmful bacteria or other pathogens enter the bloodstream, potentially leading to serious, life-threatening complications if not promptly treated. Fever, chills, hypotension, tachycardia, and altered mental status are common symptoms of the immune response triggered when an agent circulates in the bloodstream. As a result, BSIs may cause septic shock, sepsis, and eventually death. The occurrence of BSIs and the ensuing development of sepsis and septic shock are caused by several well-known risk factors. Over 90% of bloodstream infections (BSIs) are bacterial, with common pathogens including *Escherichia coli*, *Klebsiella pneumoniae*, *Pseudomonas aeruginosa*, *Staphylococcus aureus*, coagulase-negative staphylococci, and *Enterococcus* spp. Many of these, particularly ESKAPE pathogens, carry multidrug resistance, complicating treatment. Sequencing-based diagnostics face challenges in detecting these pathogens due to high genomic variability, plasmid-mediated resistance, and low pathogen load in the bloodstream. High-resolution approaches such as whole-genome or targeted deep sequencing are often required to accurately identify both species and resistance determinants [1,2].

BSIs are classified based on their origin. Community-acquired BSIs occur within 48 h of hospitalization in patients without recent healthcare exposure. Healthcare-associated BSIs arise in hospital or long-term care settings, while hospital-acquired BSIs, including intensive care unit (ICU)-acquired infections, are identified more than 48 h after admission. About 20% of ICU-acquired cases and 40% of community-acquired/hospital-acquired sepsis and septic shock cases were attributed to BSIs [3]. BSIs can be caused by a variety of factors, including surgical procedures, medical devices, such as catheters, local infectious diseases, e.g., respiratory infections, endocarditis, skin infections, urinary tract infections, sexually transmitted infections, or even both [4]. The European Centerfor Disease Prevention and Control (ECDC) reports that 8% of patients in intensive care units get a bloodstream infection, with 38% of BSI episodes being catheter related [5]. Catheter-related bloodstream infections (CRBSIs) are predominantly caused by *Staphylococcus epidermidis*, *Staphylococcus aureus*, *E. coli*, *Acinetobacter baumanii*, *Pseudomonas aeruginosa*, *Enterococcus faecalis*, and *Klebsiella pneumoniae* [6].

Sepsis diagnosis is challenging and needs to be distinguished from other sources of systemic inflammation. Accordingly, direct diagnostic proof of infection, e.g., via microbiological blood culture, is necessary for the diagnosis of sepsis [7]. The patient’s outcome depends on the prompt diagnosis of sepsis and the subsequent administration of the proper antimicrobial therapy [8]. Importantly, the patient survival rate is significantly impacted by the appropriate administration of antibiotics in the first hours following the diagnosis of BSI. Additionally, the mortality rate increases upon a delay in the initiation of the antimicrobial therapy; a 9% increase in mortality has previously been reported for each hour that a septic patient is not properly treated [9,10].

De-escalation of antibiotics, while advised, is challenging to implement in the absence of conclusive results from the gold standard diagnostic procedure. Broad-spectrum antibiotics are used as the conventional main treatment approach. Antimicrobial resistance increases as a result of this [11]. Furthermore, one in five BSI patients in U.S. hospitals are said to receive discordant empirical antibiotic therapy; this fact has been linked to higher mortality rates [12]. BSIs represent a critical global health challenge, with an estimated 48.9million sepsis cases and 11 million sepsis-related deaths worldwide [13]. These infections carry high mortality, ranging from 8% to 48% at one year, with hospital-onset and drug-resistant cases driving particularly poor outcomes [14]. Accordingly, sepsis was recently designated a global health priority by the World Health Organization [15]. Antimicrobial resistance (AMR) exacerbates the burden: in 2019, nearly 5million deaths were associated with resistant bacterial infections, including 1.27million directly caused by AMR, and resistance among leading BSI pathogens—*Esherichiacoli*, *Staphylococcus aureus*, *Klebsiella pneumoniae*, *Acinetobacter baumannii*, *and Pseudomonas aeruginosa*—is widespread [16]. Alarmingly, early projections warned that AMR could cause up to 10 million deaths annually by 2050 if unchecked [17,18]. More recent modeling estimates around 1.91 million deaths directly attributable to AMR, and 8.22 million deaths annually in which AMR is a contributing factor by 2050 [19].

### 1.2. Limitations of Traditional Culture-Based AST in Urgent Care Settings

Traditional culture-based antimicrobial susceptibility testing (AST) in urgent care settings carries several significant limitations. One of the primary challenges is the lengthy turnaround time (TAT), which delays the initiation of targeted antibiotic therapy, estimated at 24–48 h for culture. After collecting a clinical specimen, standard culture and susceptibility testing typically require 24 to 48 h to yield initial results—and up to 72 h when identification and susceptibility profiling are combined [20]. During this period, clinicians must rely on empirical broad-spectrum antibiotic therapy [21]. Not only can this delay precise treatment, but it also increases the risk of patient deterioration and may contribute to the development of antimicrobial resistance. In fast-paced urgent care environments, where patients expect immediate decisions and may not return for follow-up, this delay is particularly problematic.

Furthermore, culture-based AST is restricted by methodological constraints. Many traditional techniques, such as disk diffusion or broth microdilution, are labor-intensive and require pure bacterial isolates and manual interpretation, which can introduce variability and errors [22,23,24]. Some pathogens (e.g., anaerobes or fastidious organisms) grow very slowly or require specialized media, extending the time to actionable results. Additionally, these systems typically only test individual antibiotics, not combinations, and may fail to detect resistance mechanisms governed by complex genetic regulation or efflux pumps; mutations that are not obvious at the phenotypic level [20]. As a result, urgent care clinicians may miss atypical resistance patterns, leading to suboptimal treatment and increased risk of treatment failure. Moreover, the state of the pathogen’s viability, including viable but not culturable cells and the presence of fastidious microorganisms, can hinder bacterial growth in BC or even prevent its detection, resulting in false-negative BC outcomes [25]. Notably, blood cultures require a lengthy initial enrichment phase to reach a positive threshold due to the typically low levels of pathogens in the blood [26]. Rapid diagnostic sequencing-based platforms can substantially shorten the time to organism identification and resistance determination, allowing clinicians to initiate targeted therapy sooner and limiting unnecessary exposure to broad-spectrum antibiotics [27].

### 1.3. The Rise of Sequencing Technologies in Infectious Disease Diagnostics

The advent of sequencing technologies has substantially transformed the landscape of infectious disease diagnostics, offering a range of benefits that traditional methods could not provide. Sequencing technologies, especially next-generation sequencing (NGS), in the context of BSIs, is transforming the way we diagnose, treat, and manage these potentially life-threatening conditions. NGS enables the identification of bacteria, viruses, fungi, and parasites in a single assay, making it a powerful tool for diagnosing rare, novel, or polymicrobial infections where traditional culture and PCR fail [28]. Reports emphasize that the cost of high-throughput NGS has decreased significantly since its introduction, broadening its clinical utility from research into applied diagnostics, including in urgent care settings seeking rapid answers. In 2003, large-scale sequencing projects required investments on the order of several billion U.S. dollars, whereas current high-throughput next-generation sequencing workflows can generate a whole genome for approximately 200 U.S. dollars [29,30]. Beyond detecting pathogens, sequencing technologies offer detailed insights into antimicrobial resistance genes, virulence factors, and strain-level epidemiology [31]. Accordingly, the present review aims at evaluating and comparing sequencing platforms and technologies used in detecting AMR in BSIs, focusing on clinical sensitivity and published performance metrics.

## 2. Overview of Sequencing Technologies for AMR Detection

The rise of sequencing technologies has substantially improved the detection of AMR, offering deeper insight into its genetic basis and enabling more accurate, real-time surveillance. NGS and related genomic techniques provide rapid, high-resolution analysis, detecting resistance genes, mutations, and genomic alterations critical for AMR monitoring and personalized treatment. NGS allows the parallel sequencing of millions of DNA fragments, enabling the comprehensive detection of resistance-related genes, mutations, and structural variations in pathogen genomes. Three key approaches—whole-genome sequencing (WGS), targeted next-generation sequencing (tNGS), and metagenomic next-generation sequencing (mNGS, or clinical metagenomics)—show considerable promise for diagnosing infectious diseases. These strategies offer several advantages over conventional microbiological methods, but they also come with challenges. Each method will be assessed in terms of accuracy, turnaround time, cost, advantages, and limitations [31].

### 2.1. Whole Genome Sequencing (WGS)

WGS has become an increasingly practical clinical tool, offering high-resolution characterization of bacterial pathogens within 24–72 h after culture, depending on workflow. Its clinical value lies in the ability to rapidly identify AMR determinants, often with greater precision than conventional phenotypic AST [32]. Additionally, machine-learning techniques in conjunction with information from a bacterial pathogen’s complete genome and/or resistome (all AMR genes) have made it possible to predict the phenotypic susceptibility profile with an accuracy comparable to that of conventional growth-based techniques [33]. By interrogating established AMR-gene databases, such as CARD, ResFinder, and MEGARes, laboratories can interpret resistance markers directly from WGS data and generate resistance profiles that frequently correlate well with growth-based AST results [34]. The implementation of rapid WGS supports near–real-time infection prevention by identifying transmission events of pathogens such as *Clostridium difficile* and Methicillin-resistant *Staphylococcus aureus* (MRSA), fundamentally changing hospital outbreak management [35].

### 2.2. Targeted Sequencing Panels

Using targeted next-generation sequencing (tNGS), the area of interest, typically a gene common to all members of a microbial kingdom, is amplified directly from a clinical sample before sequencing. The sequenced amplified products enable the detection and identification of the composition of the specific microorganisms present in the sample. This method is informally known as “broad range” PCR combined with sequencing, and a prevalent illustration is the targeting of the 16S rRNA gene to enable the profiling of specific bacterial species from clinical samples. Nonetheless, it may also be utilized in a comparable way with different targets such as fungi and mycobacteria. In a broader context, extensive tNGS panels can evaluate numerous genes simultaneously, addressing pathogens and AMR targets with improved sensitivity, because of the focused design of the assays directly from patient samples. Crucially, curated microbial genome databases provide the foundation for analyzing and interpreting other direct-from-specimen sequencing methods. Consequently, the precision of tNGS and metagenomic next-generation sequencing (mNGS) frequently depends on the necessity for high-quality microbial whole-genome sequences [36].

### 2.3. Shotgun Metagenomic Sequencing (mNGS)

In contrast to tNGS, mNGS does not necessitate a suspected target and gains most of its value from an untargeted, often referred to as “shotgun” method for identifying pathogens. mNGS consists of sequencing all nucleic acids found in a sample, encompassing those sourced from the host, microbes, and even contaminating nucleic acid. Methods to eliminate undesirable nucleic acids before sequencing or unwanted reads after sequencing can be utilized, yet ultimately this approach enables the direct identification of pathogen reads amidst a vast array of total sequencing data. Consequently, mNGS is an approach that does not rely on specific hypotheses to identify all potential pathogens (such as bacterial, viral, fungal, and parasitic) simultaneously in a sample. mNGS has achieved initial success in identifying uncommon pathogens lacking targeted diagnostic tests, in recognizing pathogens that present in atypical ways, finding pathogens impacted by immunocompromising conditions that hinder routine testing effectiveness, and in the earlier identification of elusive or insidious organisms [30,37]. However, challenges persist, including the dominance of host DNA, which can account for over 90% of total sequencing reads, the extremely high cost and bioinformatic demands required for true metagenomic applications in bloodstream infections, and the need for specialized techniques like host DNA depletion or cfDNA extraction to detect pathogens in cases of low bacterial load [38]. Currently, the most extensively researched and accessible mNGS assays target pathogens in cerebrospinal fluid (CSF) and plasma, backed by numerous cases, and observational studies endorsing its application [39,40].

### 2.4. Long-Read Sequencing (Nanopore, PacBio)

Long-read sequencing technologies, such as Oxford Nanopore and Pacific Biosciences (PacBio), represent a significant leap forward in genomics, offering unique advantages in certain applications, particularly for structural variant detection, metagenomics, and AMR profiling. These technologies enable the production of much longer sequencing reads compared to traditional short-read methods, which allows for the assembly of more complex genomes and the identification of genomic features that may be missed by short-read sequencing. Therefore, the advent of long-read sequencing methods has made genome reconstruction easier and enhanced assembly continuity [41,42]. Long-read sequencing can accurately detect large-scale structural variations (e.g., insertions, deletions, inversions), which are often invisible to short-read methods. This is especially valuable in viral or bacterial strain typing and understanding gene rearrangements [43].

These technologies have transformed genomics research by allowing the examination of extensive repetitive areas, bridging gaps in current reference assemblies, and aiding in the identification of structural variations (SV), many of which are associated with different diseases. On the other hand, various initial constraints, such as restricted yield, elevated error rates, and high base costs, have obstructed the extensive use of these third-generation sequencing technologies in significant sequencing initiatives. Nonetheless, major advancements in recent years have alleviated these limitations, resulting in considerable decreases in error rates and enhancements in overall performance [44]. Oxford Nanopore and PacBio provide reads that range from 50 kilobase pairs (kbp) up to 2.3 million base pairs (Mb). The raw base-called error rate has reportedly been reduced to less than 1% for PacBio and less than 5% for nanopore sequencing, demonstrating the tremendous improvement in base-calling accuracy in both of these technologies [45].

## 3. General Workflow and Principles for Each Method

NGS revolution began in 2005 when 454 Life Sciences introduced pyrosequencing technology. This advanced technology facilitated the production and identification of thousands to millions of short reads in one machine cycle without requiring cloning. Since that time, numerous other NGS technologies have arisen that produce both short (50–400 bp) and long reads (1–100 kb). Short-read technologies that are presently utilized are commonly called massively parallel sequencing and are frequently known as second generation sequencing. They generate billions of nucleotide sequences in every run, sequencing each genome several times in small random fragments to create extremely large datasets. Despite variations in platform biochemistry and arrangements, the workflows consist of comparable steps: (i) DNA extraction; (ii) library preparation, generally involving DNA shearing via mechanical or enzymatic methods, adding adaptors and barcodes/indexes, and amplification; (iii) template preparation, through either bridge amplification or emulsion PCR; and (iv) automated sequencing [46]. Initially, extraction and purification of nucleic acids from the input tissue sample are necessary to isolate the DNA intended for sequencing. The quantity of sample required for DNA extraction differs based on the sequencing application and the type of tissue. The extracted DNA is assessed for quality, yield, and concentration, to its suitability for sequencing. Library preparation begins with the fragmentation of extracted genomic DNA into smaller fragments suitable for sequencing, achieved either through mechanical shearing or enzymatic digestion. Both ends of these fragments are ligated with short adapter sequences (oligomers), generating constructs referred to as inserts. A size selection step typically follows to ensure a uniform insert size appropriate for the intended NGS platform and to minimize the presence of adapter dimers. The resulting DNA library is then amplified using PCR to increase the overall DNA concentration prior to sequencing. Two principal strategies for targeted sequencing include hybridization capture and amplicon-based enrichment. In hybridization capture, custom-designed oligonucleotide probes (baits) selectively bind to complementary sequences within the inserts to enrich target regions. In contrast, amplicon-based enrichment uses PCR primers that flank specific genomic loci to amplify regions of interest. For increased throughput and cost-efficiency, multiple libraries can be pooled and sequenced simultaneously in a process known as multiplexing. To retain sample identity during sequencing, unique short oligomer sequences, referred to as sample barcodes or indices, typically 8–12 nucleotides in length, are incorporated into each library. These indices facilitate demultiplexing, enabling accurate assignment of sequencing reads to their respective source samples [47,48,49].

Short-read sequencing technologies vary significantly in their design, sequencing methods, output (read length, sequence quantity), precision, and cost. The Illumina platform, which presently holds a large share of the NGS market, relies on sequencing by synthesis of the complementary strand along with fluorescence-based identification of reversibly blocked terminator nucleotides. The platform features several tools that offer different throughput and read lengths [50].

Due to the tendency of short reads from second-generation sequencing platforms to produce fragmented genome assemblies, longer reads are preferable for creating closed reference genomes. These technologies specifically aim at individual DNA molecules without requiring PCR amplification. The PacBio RSII system, offered by Pacific Biosciences, utilizes single molecule real-time (SMRT) sequencing technology. Long-read sequencing relies on synthesis using nucleotides tagged with different fluorescent dyes, but the sequencing takes place when single-stranded DNA fragments are placed into small wells containing a single immobilized DNA polymerase molecule. Very lengthy DNA fragments of 20 kb or more can be achieved with run times of just a few hours [51].

### 3.1. Key Definitions: Clinical Sensitivity, Diagnostic Yield, Detection of Novel Antibiotic Resistance Genes (ARGs), Turnaround Time

Key performance metrics for evaluating NGS in clinical settings in terms of infection diagnostics include clinical sensitivity, diagnostic yield, detection of novel antibiotic resistance genes (ARGs), and turnaround time. In detail, clinical sensitivity serves as an essential performance metric for diagnostic tests utilizing NGS technology. It evaluates the test’s capacity to identify a particular genetic variant, which is essential for precise diagnosis, prognosis, and treatment choices. Clinical sensitivity is an essential performance measure for NGS tests, guaranteeing their capability to consistently identify genetic variants, resulting in precise diagnoses, informed treatment choices, and improved patient results [52].

Diagnostic yield regarding NGS signifies the percentage of tests that effectively detect a genetic variant that accounts for a patient’s condition. It primarily assesses the effectiveness of the NGS test in delivering a diagnosis that is clinically significant. The diagnostic yield of NGS differs based on the application and the population examined, typically falling between 20% and 75%. Components affecting the diagnostic yield consist of the disease’s genetic and allelic diversity, criteria for patient recruitment, clinical symptoms, sequencing technologies, and laboratory procedures. Clearly defined clinical phenotypes could yield better diagnostic outcomes with focused NGS methods [53].

Identifying new Antibiotic Resistance Genes (ARGs) is a vital focus in microbiology and bioinformatics due to the increasing worldwide danger of antibiotic resistance. New ARGs are genes that have not been previously characterized, provide resistance to antibiotics, and may evade detection by standard reference-based techniques. Identifying new ARGs requires a mix of molecular techniques and bioinformatics methods, such as NGS alongside bioinformatics tools to evaluate sequencing data and detect ARGs that have not been characterized before [54].

The turnaround time (TAT) in NGS represents the entire duration from when a sample is collected to when the results are delivered. It depends on the kind of NGS being conducted (e.g., WGS, targeted panels), whether the sequencing occurs in-house or via a commercial provider, as well as the sequencing depth, bioinformatics analysis, and reporting standards. In-house NGS provides considerably quicker turnaround times than conventional send-out NGS testing. Quick in-house NGS can yield results within 24–48h, whereas send-out tests may require 2–3 weeks [55].

### 3.2. WGS of Cultured Isolates-Workflow

WGS refers to the method of sequencing and constructing the microbial genome of a target organism. These microbial genomes may signify bacteria, fungi, and viral entities. WGS of bacteria, mycobacteria, and fungi typically requires prior culture and isolation of the organism to obtain sufficient nucleic acid for extraction and downstream sequencing. This requirement presents a significant limitation for organisms that are slow-growing, difficult to culture, or unculturable under standard laboratory conditions. For viral genomes, WGS is applied by directly sequencing the sample to obtain the viral genome of interest, which will be covered later in the metagenomics sequencing section. In summary, the organism is initially taken off the plate, and the DNA is isolated. After the DNA extraction, a library is generated where the DNA of each organism is fragmented and supplied with adapters featuring unique barcodes to facilitate the multiplexing of numerous samples. These separate libraries are combined and sent to the selected NGS technology. After sequencing is finished, bioinformatics is utilized to de-multiplex the samples, followed by quality filtering and removal of adapters. Next, there are three methods to assemble the genome for identification using WGS. The initial method, known as reference-based assembly, involves aligning sequencing reads to a well-characterized reference genome to construct a consensus sequence. In contrast, de novo assembly reconstructs the genome by assembling sequencing reads into contiguous sequences (contigs) without the aid of a reference genome [56] Reference-based assembly is faster and more efficient for identifying knownresistance genes, but it may miss plasmids or mobile resistance elements that are absent in the reference strains, potentially underestimating the full spectrum of AMR in clinical isolates [57].

#### 3.2.1. Advantages and Limitations

One of the major advantages of WGS is its high resolution, enabling the detection of known and novel resistance genes, virulence factors, and phylogenetic relationships between strains. Culturing isolates prior to sequencing ensures high-quality DNA, reducing background contamination and improving assembly quality [32]. However, WGS also has limitations. It is time-consuming compared to direct-from-sample approaches, as it requires initial culturing, which may delay diagnosis and treatment in acute clinical settings (approximately 48–72 h from positive blood culture). Apart from the need for pure isolates, the requirement for bioinformatics expertise presents significant challenges, particularly in acute sepsis where rapid clinical decision-making is crucial. Furthermore, WGS detects the genetic potential for resistance, but not phenotypic expression, which necessitates complementary antimicrobial susceptibility testing [58,59].

#### 3.2.2. Clinical Performance Data

In the clinical laboratory, WGS has shown its worth in hospital infection control initiatives by identifying and monitoring outbreaks within a hospital. Available literature highlights WGS usage in monitoring outbreaks of the prevalent hospital-acquired pathogens methicillin-resistant *Staphylococcus aureus* and *Clostridium difficile*. WGS has facilitated the monitoring of outbreaks caused by severe multidrug-resistant pathogens like carbapenem-resistant *Klebsiella pneumoniae*, vancomycin-resistant *Enterococcus faecium*, and multidrug- resistant *Acinetobacter baumannii* [60]. On the other hand, WGS enables the comprehensive detection of AMR genes, offering valuable insights for both clinical management and public health interventions. Numerous studies demonstrate the potential of WGS in predicting antimicrobial resistance for traditional microorganisms like *Esherichia coli* [61], *Staphylococcus aureus* [62], *Enterococcus faecium* [63], *Pseudomonas aeruginosa* [64], and *Neisseria gonorrhoeae* [65]. Particularly for *Neisseria gonorrhoeae*, Grad et al. discovered mtrR mutations in mosaic and a novel penA allele in mosaic that confers resistance to azithromycin and cefixime, respectively [66].

WGS can quickly influence antimicrobial resistance prediction for organisms that require extended growth times or where testing for antimicrobial susceptibility is cumbersome, such as *Mycoplasma* spp. [67]. A different category of challenging organisms that WGS can aid in speeding up antimicrobial resistance detection includes slow-growing Mycobacteria, like multidrug-resistant *Mycobacterium tuberculosis* [68,69]. In a five-year retrospective study of *Mycobacterium tuberculosis* isolates (2018–2022), WGS prediction sensitivities of 86.7% for isoniazid, 100.0% for rifampin, 100.0% for ethambutol, and only 47.8% for pyrazinamide, with specificities of 99.4%, 99.5%, 98.7%, and 99.9%, were reported, respectively [70]. In *Campylobacterjejuni* and *C. coli*, comparison of WGS-predicted AMR genes to phenotypic results yielded 97.5% concordance across five antibiotics, with only 0.6% of total isolate/antimicrobial combinations showing discrepancies [71]. WGS can notably reduce the time required for conventional antimicrobial susceptibility testing. A team has confirmed and deployed a WGS test to forecast this inducible resistance to clarithromycin along with resistance to amikacin in just 3 to 5 days, in contrast to the typical 14 days [72]. Therefore, the reduced turnaround time will help providers in managing these challenging cases.

Two beneficial uses of WGS in microbiology are resistome assessment and strain-level differentiation. Resistome denotes the complete collection of ARGs present in a sample, including those that are chromosomal, plasmid-associated, or part of a microbial community (such as the gut microbiome or a hospital setting). Conversely, strain-level resolution allows for differentiation of strains within a single species, making it possible to monitor infections, comprehend transmission, and identify pathogenic as opposed to commensal strains. Specifically, these techniques are employed to explore the origin and dissemination of resistant strains in healthcare settings or communities, track AMR in clinical or environmental samples, analyze commensal and pathogenic resistomes with high resolution, and discover new resistance mechanisms or possible therapeutic targets [73,74].

#### 3.2.3. Major Platforms & Companies

NGS today centers on four major platforms, each offering distinct advantages: Illumina, Ion Torrent, PacBio Biosciences and Oxford Nanopore Technologies (ONT). Illumina dominates short-read sequencing, leveraging sequencing-by-synthesis (SBS) chemistry to generate highly accurate paired-end reads (typically 150–300 bp), with error rates ~0.1%; its scalability, from MiSeq to NovaSeq, makes it ideal for high-throughput, high-precision applications. For longer reads, Pacific Biosciences (PacBio) uses single-molecule real-time (SMRT) technology to produce HiFi reads of 10–20kb (with >99.9% accuracy), excellent for de novo assembly, structural variant detection, and epigenetics. Oxford Nanopore Technologies (ONT), in contrast, enables ultralong reads, from several kb to >1 Mb, in portable devices like MinION and PromethION, offering real-time sequencing with flexibility for clinical use, although with higher error rates that are continuously improving [75,76]. Importantly, the newest nanopore Q20+ long-read chemistry exhibits higher accuracy, making whole-genome sequencing more sensitive for low levels of bacteria. Indeed, the novel method allows for highly accurate and extremely fast high-resolution typing of bacterial pathogens while sequencing is still in progress [77].

### 3.3. Shotgun Metagenomic Sequencing (mNGS) Directly from Blood-Workflow and Benefits

Shotgun metagenomics (mNGS) is a high-throughput sequencing approach that analyzes all genetic material present in a sample, enabling the comprehensive identification and functional characterization of microbial communities without the need for prior culturing [78]. Additionally, mNGS enables the identification of virulence genes and resistance markers, and it may yield valuable insights into molecular epidemiology [79,80]. The high costs, lengthy sequencing times, and complex data analysis historically made it impractical to use mNGS technology and bioinformatics tools on a routine basis in diagnostic labs [81]. However, improvements in workflow, including faster protocols, more affordable sequencing reagents, and streamlined analysis pipelines, have significantly reduced both time-to-result (from days to under 24 h in some cases) and cost, while easing bioinformatics burden [82].

Metagenomics is increasingly being used as a new diagnostic method for infectious diseases using two methods: targeted-amplicon metagenomics and shotgun metagenomics. In short, targeted-amplicon metagenomics is a more biased approach to a certain group of microorganisms, while shotgun metagenomics aims to sequence all the genetic material found in a sample. Amplicon sequencing focuses on conserved marker genes (e.g., 16S, ITS), which increases sensitivity for those groups but limits detection beyond bacteria or fungi. In contrast, shotgun sequencing captures all microbial DNA, including viruses, bacteria, fungi, and parasites, enabling broader detection. Shotgun metagenomics, which attempts to amplify the entire genomes of every organism found in a material, is less taxonomically biased and has a higher taxonomic resolution than the other technique. As such, it makes it possible to characterize the microbial community in more detail, including subtypes, AMR, and the carriage of pathogenic genes [83,84]. Shotgun metagenomics does have certain challenges, though. For instance, depending on the type of biological sample, a far larger amount of host DNA is frequently sequenced than the tiny portion of microbial DNA. As a result, obtaining sufficient sequencing coverage for microorganisms of interest in specimens with a large abundance of host cells might be challenging. Additionally, shotgun metagenomics is substantially more expensive than target-amplicon sequencing, depending on the necessary sequencing depth [84,85].

A typical shotgun metagenomics workflow for blood-based pathogen detection starts with host DNA depletion (e.g., saponin treatment or commercial kits) to reduce human DNA background and enrich microbial DNA. Extracted DNA is then quantified and prepared for sequencing through fragmentation and adapter ligation on platforms like Illumina or Oxford Nanopore. Sequencing, often completed within 9–12 h, produces data that undergo quality filtering, human-read subtraction, and taxonomic classification using tools such as Kraken 2, MetaPhlAn, LMAT, or GOTTCHA. Reads may be assembled into contigs or MAGs and assessed for antimicrobial resistance or virulence genes. Final outputs include pathogen identification, abundance, genome coverage, and detected AMR markers [86,87,88].

#### 3.3.1. Technical Challenges

Shotgun metagenomics offers comprehensive insights into microbial communities but comes with significant technical challenges related to contamination and interpretation. Therefore, results must be assessed in a clinical setting due to metagenomics ability to detect pathogen nucleic acid in an untargeted manner. Contaminated nucleic acids can enter the process at multiple stages during sample collection, handling, or from assay reagents and may lead to misleading results. This is particularly problematic because many common laboratory contaminants (e.g., *Staphylococcus* sp., *Pseudomonas* spp., and *Enterobacterales*) are also true pathogens, confounding interpretation. Experimental controls can help identify and mitigate reagent-based contaminants, but computational contamination including chimeric reads or non-target DNA inadvertently assembled into genomes remains difficult to detect. For instance, human DNA sequences have been identified in over 2250 bacterial genomes, creating thousands of spurious protein entries [89]. Conversely, fragments of microbial DNA can be misclassified as human and filtered out. This bidirectional misclassification underscores the need for accurate reference genomes and rigorous computational workflows for host-read subtraction and contaminant filtering [90]. It is critical to acknowledge that metagenomic NGS may generate false-positive signals from environmental or reagent contamination (e.g., *Cutibacterium acnes*, *Pseudomonas* spp.), which, if not confirmed by culture or targeted PCR, have in some clinical reports led to misdiagnoses; furthermore, because mNGS detects nucleic acids rather than viable organisms, such findings underscore the important “interpretation challenge” that signal does not always equate to active infection [31].

A major challenge in sequencing clinical specimens such as whole blood is the background of host DNA. Even after host depletion steps, human DNA can account for over 95% of sequencing reads, often leaving less than 1% available for microbial detection. This necessitates deep sequencing to recover meaningful pathogen-specific signals, significantly increasing both cost and computational demands. Cell-free DNA (cfDNA) metagenomic approaches for bloodstream infection diagnosis have been developed, but they lack genome completeness, cannot reliably detect AMR genes, and are susceptible to false positives from transient microbial DNA not linked to active infection [86,91]. While direct shot from whole blood could theoretically detect both pathogens and AMR markers, current host-depletion methods are insufficiently sensitive or fast for routine diagnostics, leading to reduced analytical performance compared to assays using other specimens like sputum [86]. Moreover, WGS sensitivity in bloodstream infections varies substantially, ranging from approximately 40% to 80%, and is strongly influenced by factors such as pathogen load, host DNA background, and the stringency of contamination control measures [92].

Finally, interpreting metagenomic results is hindered by biological and technical ambiguities. DNA-based methods cannot distinguish live from dead organisms; sequencing can detect DNA from non-viable cells or extracellular DNA, which may significantly mislead clinical interpretation, especially in low-biomass samples. Limit of detection thresholds may still miss clinically relevant pathogens, and incomplete or low-quality reference databases increase the risk of erroneous taxonomic or AMR gene assignments [93]. Indeed, commercial molecular panels have produced false positives due to unrecognized nucleic acid contaminants in blood culture media; for example, multiplex PCR panels misidentifying *Candida tropicalis* in blood cultures because of medium-derived yeast DNA, even when culture and Gram stain were negative [94]. Given these complexities, metagenomic findings should always be confirmed with conventional methods, such as culture, Gram stain, or serological testing, and interpreted alongside clinical context and other laboratory evidence to avoid misdiagnoses. Ultimately, in clinical applications, where accuracy is paramount, these challenges underscore the need not just for updated databases but for standardized quality-control procedures and clear versioning of database builds to ensure reproducibility and reliability in pathogen detection workflows.

#### 3.3.2. Leading Commercial Platforms

Several leading commercial platforms have been developed to facilitate shotgun metagenomic sequencing for clinical and research applications, providing streamlined workflows from sample processing to data analysis. The Illumina MiSeq and HiSeq platforms are among the most widely used short-read sequencers for shotgun metagenomic analyses. These platforms are commonly integrated with bioinformatics pipelines such as DRAGEN or BaseSpace, which facilitate high-sensitivity and high-specificity detection of pathogens, identification of AMR genes, and comprehensive microbiome profiling. Despite having an accuracy range of 0.1 to 1%, Illumina sequencers can have long run durations (up to 55 h for MiSeq and 84 h for HiSeq), and their equipment costs range from moderate to high [75]. On the other hand, Oxford Nanopore Technologies (ONT) has also emerged as a disruptive force with its portable MinION and larger GridION and PromethION devices, which enable real-time sequencing and rapid turnaround times. ONT’s platforms excel in long-read sequencing, offering advantages for resolving complex genomic regions and structural variants, although with somewhat higher raw read error rates compared to Illumina The MinION technology is small and has the potential for a quick turnaround time of a few hours from DNA extraction to the acquisition of sequence data [95]. Before being widely used, several issues must be resolved, such as sequencing accuracy, the creation of reliable analytical pipelines for long-read sequencing, and increased sequencing costs [96].

#### 3.3.3. Performance Metrics

Shotgun metagenomics enables the comprehensive detection of AMR genes by sequencing all genetic material in a sample, providing a high breadth of coverage across diverse microbial communities. The sensitivity of AMR gene detection in this approach depends largely on sequencing depth, sample complexity, and bioinformatic pipelines used for identifying resistance determinants. Higher sequencing depths increase the likelihood of capturing low-abundance AMR genes, thereby improving detection sensitivity, especially in samples with diverse or rare resistance elements. Moreover, advanced bioinformatic tools that leverage curated AMR gene databases such as CARD or ResFinder further enhance sensitivity by accurately annotating resistance genes amidst vast metagenomic data [97]. As was previously reported, a high-throughput nanopore sequencing workflow was developed for detecting pathogens in cell-free DNA from plasma, achieving real-time identification of pathogens within 5–6 h, with over 90% concordance with standard sequencing results in a cohort of septic patients [98].

In addition to sensitivity, the breadth of AMR gene detection in shotgun metagenomics is a key advantage over targeted methods, as it allows for the identification of known and novel resistance genes across multiple antibiotic classes in a single assay. However, the breadth may be limited by incomplete reference databases or the presence of highly divergent AMR genes that escape annotation. Continuous updates to AMR gene repositories and integration of machine learning approaches for novel gene discovery are advancing the breadth and accuracy of resistance gene detection in metagenomic studies [99].

#### 3.3.4. Turnaround Time and Real-World Diagnostic Impact

One of the major advantages of shotgun metagenomics is the significantly reduced turnaround time compared to traditional blood cultures, which often require 24 to 72 h or longer for pathogen identification. Metagenomic sequencing can deliver actionable diagnostic results within 24 to 48 h, which is critical for initiating targeted antimicrobial therapy in septic patients. This rapid turnaround can potentially improve clinical outcomes by facilitating early and precise pathogen identification, especially in cases where conventional methods fail due to slow-growing, fastidious, or unculturable organisms. Test results are acquired in 1–2 days using Oxford Nanopore’s rapid sequencing technique compared to short-read sequencing that has a longer turnaround time [88,100,101,102]. For taxonomy identification tests, the sequencing run time onto the MinION flow cell (R9.4.1, Oxford Nanopore Technologies) is roughly 3 h, and for AMR experiments it is between 3 and 24 h [86].

In real-world clinical settings, shotgun metagenomics has shown substantial diagnostic impact by increasing the detection rate of bloodstream pathogens, guiding more effective treatment strategies, and reducing empirical broad-spectrum antibiotic use. Its comprehensive detection capability not only identifies bacteria but also viruses, fungi, and parasites, providing a more complete infectious disease profile. Furthermore, metagenomic data can inform antimicrobial resistance gene presence, supporting precision medicine approaches [30].

In addition, shotgun metagenomics is a useful tool for identifying acute intra-abdominal infections (IAIs) in sepsis patients, particularly in culture-negative cases, since studies show that it offers higher sensitivity and a wider pathogen detection range in plasma when compared to traditional culture methods [103]. The use and duration of broad-spectrum antibiotics like carbapenems and anti-MRSA treatments can be decreased by more focused and efficient antibiotic therapy prompted by fast and thorough pathogen detection provided by shotgun metagenomics. All things considered, the clinical care and microbiological diagnosis of sepsis and acute IAIs could be greatly improved by combining metagenomics with traditional diagnostic techniques. This would improve patient outcomes and maximize the use of antibiotics [103]. Notably, a pathogen’s virulence is another crucial aspect that clinicians may consider when deciding on the best course of action. Early detection of virulence factors is crucial as horizontally acquired virulence genes can directly cause an infectious outbreak [104].

#### 3.3.5. Limitations and Gaps in Validation

Despite its promise, mNGS faces several limitations and gaps in validation that hinder its routine clinical implementation. One major issue is the lack of standardized protocols for sample processing, sequencing platforms, and bioinformatics pipelines, which can lead to variability in results across laboratories [105]. Additionally, challenges in distinguishing true pathogens from background contamination or commensal organisms complicate clinical interpretation, particularly in low microbial biomass samples like blood [80]. Additionally, pathogen quantity, specimen composition, and AMR determinant type all affect mNGS’s capacity to detect AMR. In contrast, low-abundance pathogens often yield fragmented genomes in mNGS, limiting genome coverage and resulting in under-detection of AMR markers, even when pathogen presence is confirmed [31]. While pathogen identification via mNGS can succeed with a few unique reads, reliable AMR gene calling requires substantially more data: studies show that around 15× genome coverage (i.e., hundreds of thousands to millions of reads) is generally necessary to detect resistance genes accurately for moderate-to-high abundance pathogens; detecting AMR in organisms comprising ~1% of the sample may demand tens of millions of reads [106]. Another significant limitation is the insufficient validation of metagenomic tests in large, prospective clinical trials, making it difficult to assess sensitivity, specificity, and clinical utility in real-world settings [80]. These gaps highlight the urgent need for consensus guidelines, robust analytical validation, and multi-center clinical studies to establish metagenomics as a reliable diagnostic tool.

### 3.4. Targeted Sequencing Panels

For purposes of diagnosis, prognosis, treatment monitoring, etc., targeted sequencing, often referred to as pan-bacterial (e.g., based on 16S rRNA) or pan-fungal (e.g., based on ITS) targeted amplicon deep sequencing, focuses on a limited number of genes. Consequently, utilizing tailored sequencing panels in clinical settings can lower costs while increasing confidence and improving insurance reimbursement opportunities [107]. By incorporating an enrichment method for microbial sequences of interest before library construction, targeted NGS (tNGS) improves analytical sensitivity. Prior to sequencing, highly conserved sections of bacterial or fungal DNA are amplified in the most used enrichment technique for clinical applications. For example, the 16S ribosomal RNA gene, which is conserved in all bacteria, is targeted and amplified by primers in tNGS for bacterial identification [108]. Viral genome alterations, particularly those associated with resistance in viruses like HIV, hepatitis B, and CMV, can be detected directly from clinical specimens with excellent sensitivity thanks to the enrichment process. By amplifying the target’s nucleic acids to millions of copies, the enrichment stage dramatically increases the quantity of target-specific sequencing reads. This is in contrast to mNGS, which improves the sensitivity and precision of pathogen detection by obtaining the bulk of sequence reads from the host genome [107]. Illumina’s targeted amplicon sequencing library preparation begins with an initial PCR amplification step to generate amplicons of the desired genetic marker, incorporating adapter sequences in the process. Sequencing primers and sample-specific barcodes are subsequently added, followed by library quantification and normalization prior to sequencing [96].

Hybrid capture-based targeted NGS offers an alternative approach that uses biotinylated oligonucleotide probes to enrich known AMR genes across complex microbial communities. This method improves the detection of low-abundance resistance genes and enables broader genome coverage, making it particularly effective in metagenomic samples such as blood. Hybrid capture panels, such as those developed from the ARESdb or QIAseqxHYB AMR designs, have demonstrated substantial increases in the proportion of AMR-related reads, reaching up to several thousandfold enrichment compared to untargeted sequencing. These panels can identify a wide range of resistance mechanisms, including extended-spectrum beta-lactamases (ESBLs), carbapenemases, and plasmid-mediated colistin resistance (*mcr* genes), often in polymicrobial or low-biomass samples. While hybrid capture retains the limitation of database dependence, its improved sensitivity and ability to capture flanking genomic regions provide valuable context for understanding gene mobility and resistance transmission [109].

#### 3.4.1. Benefits

Targeted NGS offers a powerful, rapid, and sensitive approach for diagnosing bloodstream infections compared to traditional blood culture and even untargeted metagenomic sequencing. By using amplification or hybrid capture to enrich for clinically relevant pathogens and resistance genes, tNGS achieves much higher sensitivity; sensitivity up to 91% versus just ~23% for blood culture and ~70% for mNGSis reported [110]. In a cohort of 387 samples, nanopore-targeted sequencing produced results within 7 h, detected similar pathogen positivity rates to metagenomics, and correlated genotypic AMR prediction with phenotypic resistance in over 80% of cases [111]. This rapid turnaround, typically under 24 h and sometimes as fast as 6 h, drives earlier targeted antibiotic therapy, accelerates de-escalation, and helps identify co-infections and hard-to-culture organisms that standard cultures miss [112]. Although tNGS has a smaller microbial detection spectrum than shotgun mNGS, it is more affordable and less susceptible to host/background DNA interference, making it a desirable choice for pathogen identification straight from clinical samples. Overall, tNGS strikes an optimized balance of speed, diagnostic accuracy, and cost-efficiency, making it highly suitable for clinical deployment in BSI diagnosis and antibiotic stewardship efforts.

#### 3.4.2. Limitations

Targeted NGS approaches offer significant advantages by enriching specific AMR markers, but their reliance on predefined panels also imposes important limitations. Since these assays use primer or probe sets tailored to known genes, they inherently cannot detect novel or highly divergent resistance determinants not included in the panel, leading to potential false-negative results, especially concerning as AMR evolves rapidly [31]. This panel dependency means that any emerging gene variants or resistance mechanisms that arise post panel design will be overlooked unless the panel is frequently and rigorously updated to keep pace with microbial evolution.

Another challenge with predefined panels is that they may fail to capture resistance emerging from point mutations or structural variations outside targeted regions. Unlike whole-genome sequencing, which can reveal unknown determinants, targeted NGS often misses AMR mediated by single-nucleotide polymorphisms (SNPs) or mutations in promoter or regulatory regions because these regions are outside the scope of typical probe designs. Targeted sequencing outcomes reflect only the presence or absence of antibiotic resistance-associated genetic elements in a sample; therefore, clinical context and phenotypic data are typically required to accurately interpret these results. Furthermore, in polymicrobial or complex samples, enriching only for selected gene targets may skew the relative abundance of organisms and obscure the broader resistome context, complicating the interpretation of mixed infections [113]. Thus, while targeted NGS offers high specificity and sensitivity for known genes, it requires complementary methods, such as untargeted metagenomics or frequent panel revisions, to avoid missing emerging or unexpected resistance mechanisms.

#### 3.4.3. Prominent Commercial Assays

Several commercial targeted NGS panels for infection diagnostics, emphasizing AMR detection and broader pathogen profiling, are available. A prominent example is the Illumina™ AmpliSeq™ Antimicrobial Resistance Research Panel, which employs AmpliSeq™ technology to target a comprehensive set of 478 known AMR genes spanning 28 antibiotic classes. This panel utilizes two primer pools to generate 815 amplicons, enabling broad and efficient coverage of clinically relevant resistance determinants. This panel enables rapid and accurate identification of resistance determinants in both Gram-positive and Gram-negative bacteria, such as *Staphylococcus aureus*, *Escherichia coli*, *Klebsiella pneumoniae*, *Pseudomonas aeruginosa*, and *Enterococcus* species, by delivering quick, cost-effective results compatible with Illumina MiSeq and NextSeq platforms. It includes well-characterized genes conferring resistance to β-lactams, aminoglycosides, fluoroquinolones, tetracyclines, carbapenems, macrolides, and vancomycin, providing broad-spectrum coverage critical for clinical decision-making. The panel’s integration with Illumina sequencing platforms, including MiSeq™ and NextSeq™, facilitates high-throughput processing with rapid turnaround times, often delivering results within 24 h, making it particularly valuable in clinical settings where timely resistance profiling can guide targeted therapy and support antimicrobial stewardship. Its utility is amplified in bloodstream infection scenarios, where identifying the resistance profile quickly can significantly improve patient outcomes and reduce reliance on empirical broad-spectrum antibiotics. This compatibility and efficiency have made the AmpliSeq AMR Panel a powerful tool for modern clinical microbiology and resistance surveillance efforts [114].

The Thermo Fisher Ion AmpliSeq™ AMR Panel is designed for the detection of AMR genes in bacterial pathogens, enabling the precise identification of resistance profiles in clinical microbiology and research settings. This panel is based on Ion AmpliSeq™ technology, which leverages NGS technology to detect and analyze multiple AMR-associated genes simultaneously. Key genes targeted by the Ion AmpliSeq™ AMR Panel include β-Lactam Resistance Genes (blaTEM, blaSHV, blaCTX-M, blaOXA, blaKPC), Aminoglycoside Resistance Genes (aac(3)-IV, aadA1, strA/strB), Fluoroquinolone Resistance Genes (gyrA, parC), Tetracycline Resistance Genes (tetA, tetB), Macrolide Resistance Genes (ermB, mefA), Vancomycin Resistance Genes (vanA, vanB), Sulfonamide Resistance Genes (sul1, sul2), and Other Resistance Genes (mecA, tetM). The Ion AmpliSeq™ AMR Panel detects resistance markers across a range of bacterial pathogens, including Gram-negative bacteria (*Esherichia coli*, *Klebsiella pneumoniae*, *Pseudomonas aeruginosa*, *Acinetobacter baumannii*, *Salmonella* spp.) and Gram-positive bacteria (*Staphylococcus aureus* (including MRSA), *Enterococcus faecium* and *Enterococcus faecalis*, *Streptococcus pneumoniae*, other important pathogens (*Mycobacterium tuberculosis*, which has resistance to rifampin and isoniazid). Ion AmpliSeq™ technology is based on targeted sequencing, where specific regions of the genome are selectively amplified. This method involves designing specific primers for resistance-related genes, which are then amplified using PCR. The prepared libraries are subsequently sequenced using ion semiconductor sequencing technology, which is recognized for its rapid turnaround time and high accuracy in DNA sequencing. This platform offers high-throughput capabilities and enables real-time data generation and analysis, making it well-suited for time-sensitive applications such as pathogen detection and antimicrobial resistance profiling [115].

The Oxford Nanopore MinION allows researchers and clinicians to design custom panels targeting specific AMR genes or genetic regions. This is a highly flexible approach, as users can focus on the resistance genes relevant to their region, the population of pathogens they are dealing with, or emerging resistance patterns. Custom panels can be tailored to cover resistance mechanisms for antibiotics such as β-lactams, aminoglycosides, tetracyclines, fluoroquinolones, macrolides, and others [116]. A custom AMR panel consists of primers designed to target specific AMR genes and resistance-associated sequences across a broad range of pathogens. After extracting bacterial DNA from clinical or environmental samples, the custom AMR panel primers are used to selectively amplify the resistance genes of interest. This step often involves using PCR-based amplification followed by library preparation for nanopore sequencing. Libraries are prepared by adding adapter sequences to the amplified fragments, which are then ready for sequencing with the MinION platform. The MinION platform sequences the prepared libraries by reading DNA molecules as they pass through nanopores, during which changes in electrical current are measured and translated into nucleotide sequence data. The long-read capability of nanopore sequencing enables the resolution of large genetic regions, operons, and gene clusters, and facilitates the detection of structural variations and novel mutations that may confer antimicrobial resistance [117].

#### 3.4.4. Comparative Performance of tNGS

Targeted NGS for BSIs offers strikingly improved sensitivity, coverage, usability, turnaround time, and workflow integration compared with traditional culture or mNGS approaches. Its sensitivity is significantly enhanced by ultra-multiplex PCR enrichment and host-DNA removal. In a study of 387 BSI samples, nanopore-based tNGS showed 84.0% sensitivity and 90.1% specificity, compared with conventional blood culture’s 33.9% positivity [111]. The coverage of tNGS is similarly broad: panels routinely target over 300 clinically relevant bacteria, fungi, and viruses, including resistance and virulence markers, enabling precise species and subspecies identification. In contrast to mNGS’s complex demands, tNGS is more usable in clinical labs—requiring moderate sequencing depth, lower data volume, fewer bioinformatics resources, and often leveraging existing mid-throughput instruments. The time-to-result is significantly shorter: results may be available within 12–24 h, with some protocols delivering actionable data in as little as 18h, far quicker than culture (2–5 days) or mNGS (36 h–3 days) [118]. Regarding cost, DNA-only tNGS is estimated at ~$490 USD per sample, while dual DNA/RNA sequencing ranges from ~$630 to $910 USD per sample [110]. Finally, workflow integration is seamless; tNGS pipelines involve rapid sample preparation, multiplexed PCR, library preparation, sequencing, and streamlined analysis, allowing incorporation into standard microbiology lab operations with minimal retraining [119,120]. Together, these features position tNGS as a faster, more targeted, and cost-effective tool for BSI diagnostics in the routine clinical setting.

### 3.5. Long-Read Sequencing (Nanopore & PacBio)-Advantages and Limitations

Long-read sequencing technologies, such as Oxford Nanopore Technologies (ONT) and Pacific Biosciences (PacBio), have significantly advanced pathogen genomics by enabling the generation of reads spanning tens to hundreds of kilobases. ONT routinely produces reads in the range of 10–100 kb, with potential for megabase-length sequences, while PacBio’s high-fidelity (HiFi) reads offer lengths of 10–20kb with exceptional accuracy. These platforms facilitate the accurate reconstruction of complex genomic regions, including structural variants, repetitive elements, plasmids, and epigenetic modifications such as DNA methylation, outcompeting short-read sequencing that cannot address this issue efficiently [44]. PacBio’s Single-Molecule, Real-Time (SMRT) HiFi sequencing now achieves a base accuracy approaching 99.9%, combining long reads with Illumina-level precision, while ONT’s latest chemistries and deep-learning base-calling are pushing raw error rates down to ~1–5%. ONT’s hallmark is real-time, amplification-free sequencing on portable platforms (e.g., MinION, PromethION), allowing for extremely rapid in-field detection of outbreaks and flexible adaptive sampling. PacBio, while less mobile, excels in high-fidelity accuracy and epigenetic profiling via SMRT sequencing. Long-read sequencing provides real-time data acquisition, allowing users to begin analysis before sequencing has finished, which is particularly valuable in time-sensitive clinical or epidemiological settings. Moreover, adaptive sampling, a distinctive feature of Nanopore sequencing, enables the real-time selective depletion of host DNA during sequencing, thereby enriching microbial sequences and improving downstream analytical sensitivity [121]. Finally, due to their extended read lengths, long-read technologies can resolve complex genomic structures, including plasmids and mobile genetic elements (MGEs), which are often challenging to characterize with short-read approaches. This capability is crucial for studying antimicrobial resistance and horizontal gene transfer [41,44].

**Figure 1:**
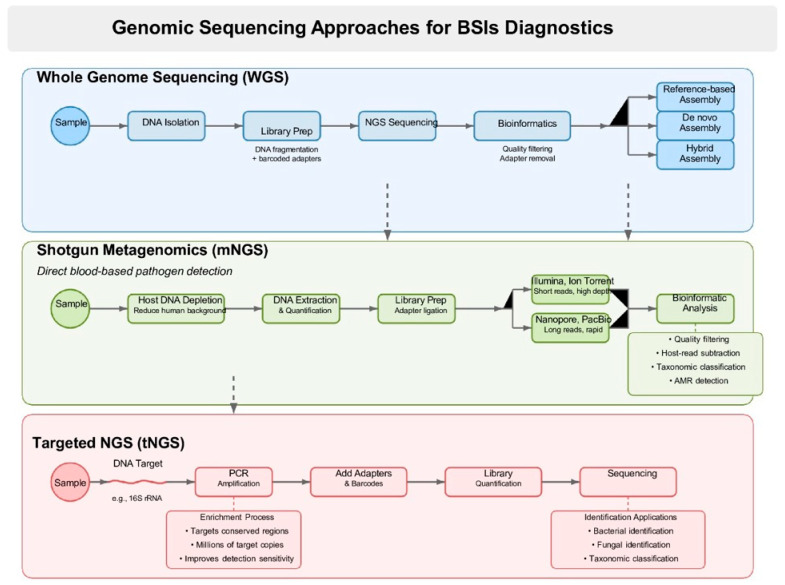
Overview of the three NGS workflows used in bloodstream infection (BSI) diagnostics. The figure illustrates whole genome sequencing (WGS), shotgun metagenomics (mNGS), and targeted NGS (tNGS) workflows. Each workflow is depicted with its sample processing steps, sequencing strategies, and downstream bioinformatic analysis.

However, long-read sequencing still faces some notable limitations. ONT, while cost-effective and portable, has historically suffered from lower per-base accuracy compared to short-read platforms (error rates ~5–15%), although recent improvements (e.g., Q20+ chemistry and R10.4 flow cells) have narrowed this gap significantly [122]. Conversely, PacBio HiFi sequencing provides high accuracy, but the technology is cost-intensive and requires more substantial infrastructure, which may limit its broader adoption in routine clinical labs, particularly in clinical microbiology facilities. Furthermore, the clinical readiness of long-read sequencing remains in development. While its potential in infectious disease diagnostics is promising, integration into clinical workflows is still limited by regulatory, cost, and data interpretation challenges [123]. Ultimately, Figure 1 provides a comparative overview of the three NGS workflows, WGS, shotgun metagenomics, and targeted NGS, highlighting their respective approaches and applications in the diagnosis of BSIs.

#### 3.5.1. Pilot Studies and Proof-of-Concept Trials in BSI and AMR Detection

Long-read sequencing has been increasingly investigated in pilot studies and proof-of-concept trials for the detection of BSIs and antimicrobial resistance. These early investigations demonstrate the feasibility of using technologies like Oxford Nanopore and PacBio for rapid pathogen identification directly from clinical samples, often without the need for culture. For example, Charalampous et al. conducted a pilot study utilizing nanopore metagenomic sequencing to detect pathogens in respiratory and bloodstream infections, showing that clinically relevant organisms could be identified within hours [116]. More recently, a dedicated Nanopore-based protocol (PISTE) reported a total “time to result” of roughly 6.5 h compared to ~30.4 h for standard-of-care blood-culture-based methods [124]. Liu et al. conducted a study evaluating the feasibility and performance of ONT sequencing with adaptive sampling for rapid pathogen identification and AMR prediction directly from positive blood cultures. The study demonstrated that this approach enabled accurate species identification within 1 h and comprehensive AMR profiling within 15 h, including the detection of polymicrobial infections and novel species [125]. Notably, a recent study revealed a significantly short average time cost of ONT NGS of less than 4 h [126]. These studies highlight long-read sequencing’s potential to improve infectious disease diagnostics by enabling rapid, culture-independent detection.

#### 3.5.2. Potential Future Use in Rapid Point-of-Care Diagnostics

Long-read sequencing has the potential to revolutionize point-of-care (POC) diagnostics by enabling rapid, on-site detection and characterization of pathogens and antimicrobial resistance genes directly from clinical samples. Recent advances in ONT technology, coupled with decreasing costs and faster turnaround times, have made it a desirable tool for several genomic applications. Increasingly, ONT sequencing is being used in clinical microbiology labs, especially for real-time genomic surveillance, drug resistance discovery, infectious disease research, and the identification of uncommon and unknown pathogens [127]. As recently demonstrated, nanopore-based workflows were used to perform ultra-rapid whole-genome sequencing on critically ill patients, leading to diagnoses of rare genetic diseases in approximately 8 h [128].

Portable devices like Oxford Nanopore’s MinION and upcoming all-in-one platforms (e.g., TraxION) are compact and user-friendly, making sequencing feasible in various clinical settings, including emergency rooms, and/or outpatient clinics. Importantly, as bioinformatics tools continue to evolve, incorporating artificial intelligence (AI) and machine learning (ML) to interpret complex sequencing data in real-time, the workflow for POC long-read sequencing is expected to become increasingly streamlined and automated [44,128]. Therefore, such rapid diagnostics will be invaluable for controlling outbreaks, managing sepsis, and tailoring antimicrobial therapy.

## 4. Comparative Summary: Table/Matrix: Performance Comparison of Sequencing Platforms

A comparative table/matrix (Table 1) that outlines the performance characteristics of several popular sequencing platforms, including Illumina MiSeq/NextSeq, Ion Torrent S5/Ion Proton, Oxford Nanopore MinION, and PacBio Sequel IIe HiFi is presented below. The focus is on key parameters like read length, accuracy, throughput, turnaround time, cost, strengths, and limitations.

Accordingly, Illumina MiSeq/NextSeq performs sufficiently for high-throughput sequencing with exceptional accuracy, making it the gold standard for large-scale genomic studies, clinical diagnostics, and transcriptomic profiling. On the other hand, Ion Torrent S5/Ion Proton serves as a solid choice for targeted sequencing and clinical diagnostics with moderate throughput and cost, especially when a faster turnaround is required. Regarding Oxford Nanopore MinION, the implementation of such technology is suitable for fieldwork, real-time sequencing, and custom AMR panels, especially where long reads are beneficial and rapid diagnostics are needed in remote or resource-limited settings. PacBio Sequel IIe system offers HiFi long reads with high accuracy, ideal for resolving complex genomic regions, though it involves higher instrument and per-base costs along with longer library preparation times. Moreover, a concise Table 2 highlights key findings from ICU and sepsis cohorts to better illustrate the clinical impact of sequencing on patient management and decision-making.

## 5. Regulatory and Quality Assurance Frameworks for Clinical Next-Generation Sequencing: CLSI, CAP, and ISO 15189 Guidelines

Regulatory frameworks and quality-assurance in clinical next generation sequencing (NGS) are built upon several well-established standards and guidelines to ensure accurate, reproducible, and clinically meaningful results. One cornerstone is the Clinical and Laboratory Standards Institute Guideline MM09 (CLSI MM09) guideline, Human Genetic and Genomic Testing Using Traditional and High Throughput Nucleic Acid Sequencing Methods, which provides detailed, step by step recommendations for the design, development, validation, reporting, and ongoing quality management of NGS assays. Complementing that, the College of American Pathologists (CAP) has developed NGS-specific accreditation checklists and “worksheets” that guide laboratories across the full test lifecycle—pre-analytic, analytic, and post-analytic—and are maintained via annual expert reviews [135]. On a broader scale, laboratories often seek accreditation under ISO15189, the international standard for medical laboratory competence, which requires a robust quality management system (QMS) tailored to clinical laboratories. For molecular genetic testing, specific application guidance under ISO15189 emphasizes documented QA/QC processes for all phases of the NGS workflow, including personnel training, method standardization, reference materials, and proficiency testing. Moreover, external quality assessment (EQA) under ISO15189 has been demonstrated in practice; for example, on-site evaluations of NGS oncology testing have uncovered variability in variant detection and reporting, leading to actionable improvements [136]. Finally, public health-oriented initiatives such as the CDC Next Generation Sequencing Quality Initiative provide complementary quality management tools based on the 12 Quality System Essentials (QSEs) originally defined by CLSI, helping labs build or strengthen their QMS in alignment with both accreditation and regulatory requirements [137]. Together, these frameworks create a multi-layered regulatory and quality assurance architecture that supports trustworthy and clinically actionable NGS reporting.

## 6. Clinical Considerations and Implementation

The clinical adoption of sequencing technologies for AMR detection centers on a range of interdependent factors that affect feasibility, diagnostic yield, and clinical utility. Central to this process is the selection of the most suitable sequencing platform, which should be guided by the specific clinical scenario, urgency of results, type of sample, and the intended downstream application—be it individual patient care, outbreak control, or population-level surveillance.

In settings involving acutely ill or immunocompromised patients—such as intensive care units, hematology/oncology wards, or transplant units—rapid diagnostic turnaround is often essential for guiding timely therapeutic decisions. In these cases, targeted sequencing approaches (e.g., resistance gene panels) or real-time portable platforms such as nanopore sequencing may be most appropriate [138]. These methods can deliver actionable data within hours and are particularly useful when culture is slow or inconclusive, as in the case of fastidious or unculturable pathogens. For broader genomic insights, especially in scenarios requiring strain typing, outbreak investigation, or resistance mechanism discovery, more comprehensive approaches like WGS offer unparalleled resolution. WGS enables the simultaneous detection of AMR determinants, virulence factors, phylogenetic relationships, and horizontal gene transfer elements [139]. Although its turnaround is typically longer and its bioinformatics requirements more demanding, WGS is invaluable in reference laboratory settings, epidemiological research, and retrospective analysis. Metagenomic sequencing, which allows for untargeted analysis of all genetic material in a clinical or environmental sample, provides an even broader picture. This is particularly useful in polymicrobial infections, culture-negative sepsis, or samples where the pathogen is unknown or unexpected [40,140].While powerful, metagenomics presents additional technical and interpretative challenges—such as host background interference, detection thresholds, and the need for advanced data processing pipelines—and is not yet suited for routine frontline diagnostics. Amplicon sequencing (e.g., 16S rRNA or ITS regions) offers another alternative, particularly when the goal is to identify species composition in polymicrobial infections or to detect shifts in microbial communities under selective pressure from antimicrobials. However, it has limited resolution for resistance profiling [141]. The choice of platform must also take into account the nature of the specimen (e.g., blood, sputum, urine, tissue, or environmental water sample), the prevalence and resistance profile of local pathogens, and available laboratory capacity. For instance, blood culture-positive isolates lend themselves well to WGS, while direct sequencing from sputum or stool may require metagenomics or hybrid approaches.

Finally, regulatory approval, data interpretation tools, sample throughput, and cost per test all weigh into platform selection. While high-throughput short-read sequencers (e.g., Illumina platforms) offer accuracy and multiplexing, they may not be practical for urgent bedside diagnostics. In contrast, portable long-read sequencers (e.g., Oxford Nanopore) can be deployed at the point of care but may require post-sequencing polishing or hybrid validation.

Ultimately, no single sequencing method is optimal for all clinical applications. An informed choice must balance diagnostic needs with technological capabilities, logistics, and the clinical context in which sequencing is to be deployed. Laboratory capabilities also play a central role, as not all institutions possess the infrastructure, bioinformatics support, or trained personnel required to implement and sustain sequencing workflows. Additionally, the regulatory status of each platform (e.g., CE-IVD, FDA-approved, or RUO—research use only) directly affects its applicability in routine diagnostics and influences institutional willingness to adopt these tools. Integration with ASP is crucial to ensure that sequencing-derived resistance data inform clinical decision-making and guide optimal antimicrobial therapy. Such integration enhances the precision of empirical treatment, reduces inappropriate antimicrobial use, and supports infection control interventions [142].

Cost-effectiveness is another critical dimension. While sequencing may have higher upfront costs compared to conventional diagnostics, its ability to reduce treatment failures, prevent outbreaks, and shorten hospital stays can yield downstream economic benefits. Scalability is equally important; platforms must be adaptable to both high- and low-resource settings, which often face divergent logistical, financial, and staffing constraints [143].

Despite these advantages, several barriers continue to impede widespread implementation. Reimbursement policies for sequencing-based diagnostics remain underdeveloped in many health systems, disincentivizing their routine use. A lack of standardized protocols and reference databases complicates result harmonization across laboratories. Moreover, healthcare professionals often require additional training to interpret complex sequencing outputs, and integration into electronic health records and clinical workflows is not yet streamlined.

## 7. Future Perspectives

Global efforts to tackle the AMR spreading via monitoring and new antibiotic discoveries have resulted in the accumulation of datasets of diverse bacterial genomes, AST, and chemical bioactivity screens.

### 7.1. AI-Enhanced Prediction of Phenotypic Resistance from Genotypes

Large data quantities are easily handled by AI techniques, which makes them a powerful tool for deriving insightful information from intricate datasets. A kind of artificial intelligence called machine learning (ML) makes use of statistical algorithms to find complex associations in datasets and extrapolate to new data [144]. AI has a growing and highly specific role in improving the diagnosis and management of bloodstream infections, particularly in the early detection of antimicrobial resistance [145]. By integrating genomic data generated from NGS platforms—including WGS and long-read sequencing—AI and machine-learning models can rapidly predict phenotypic resistance patterns from raw sequence data, enabling earlier optimization of antimicrobial therapy in septic patients. These algorithms are especially valuable for identifying complex resistance determinants, such as plasmid-borne or structurally embedded AMR genes, which often challenge conventional bioinformatic analysis. Beyond pathogen genomics, AI applied to electronic health records can enhance diagnostic precision by detecting biomarker and clinical patterns that distinguish infectious from non-infectious systemic inflammation, supporting more accurate early recognition of sepsis. In culture-negative or polymicrobial BSIs, where conventional diagnostics fail, AI-driven analysis of metagenomic and transcriptomic outputs can help resolve ambiguous cases and improve pathogen identification. Together, these targeted applications demonstrate that AI is not a general adjunct but a clinically relevant tool that directly strengthens sequencing-based diagnostics for BSIs by improving AMR prediction, accelerating decision-making, and supporting antimicrobial stewardship [146].

### 7.2. Real-Time Sequencing in Emergency/ICU Settings

One major key advance in ICU real-time sequencing includes metagenomic nanopore sequencing for bloodstream infections. As recently revealed, a multicenter study at Oxford screened 273 blood cultures achieving 97% pathogen identification sensitivity and 94% specificity. AMR detection was completed 20 h faster than the conventional method with results delivered in 3.5 h post-culture [147]. Moreover, rapid susceptibility testing in ICU-positive cultures is an emerging strategy. Prospective ICU evaluation in patients with suspected sepsis showed species identification accuracy of ~94% vs. standard methods. Predicted AST results for some pathogens were delivered within 8–16 h. Before this method can be considered for clinical use, it must first increase sequencing accuracy and develop more reliable predictive algorithms over a wide range of organisms, even though it showed promise and performed well for certain common bacterial species. Nonetheless, results could be obtained more quickly than with traditional phenotypic techniques [148]. As recently reported, a multicenter clinical trial (DIRECT) included 156 participants across four Brisbane ICUs to assess rapid pathogen identification via Oxford Nanopore Technologies MinION sequencing. An important preliminary outcome highlights the antibiotic dosing software usage. Indeed, among patients who had not reached therapeutic drug levels within 24 h, use of individualized dosing software shortened the time to effective drug exposure by more than 48 h [149].

### 7.3. Multi-Omic Integration (Resistome + Transcriptome + Host Response)

Multi-omics approaches can greatly strengthen pathogen and antimicrobial resistance detection in bloodstream infections by combining several molecular layers that each describe a different aspect of the host–pathogen interaction. Genomic and transcriptomic analyses characterize pathogen composition and reveal changes in gene expression that reflect immune activation, inflammatory signaling, and metabolic stress—features that help distinguish infectious from non-infectious systemic responses and guide antimicrobial decisions. Proteomic and metabolomic profiling add further resolution by capturing alterations in cytokines, chemokines, and metabolic intermediates associated with sepsis severity and progression. When these datasets are integrated, they provide a more complete picture of both microbial activity and host dysregulation, enabling earlier recognition of infection-related molecular patterns and improving the accuracy of resistance prediction. Multi-omics models also make it possible to identify patient subgroups with distinct biological signatures, which can inform treatment personalization and antimicrobial stewardship. By unifying host and pathogen signals into a comprehensive molecular profile, multi-omics has the potential to enhance the diagnostic precision of sequencing workflows and support earlier, better-targeted therapy in bloodstream infections.

Multi-omic integration is the approach of combining data from multiple omics layers, such as resistome, transcriptome, and host response, to provide a comprehensive and holistic view of disease mechanisms, especially in complex diseases like infections and sepsis. Integrating these layers of data can lead to a deeper understanding of how pathogens interact with the host and how AMR evolves and spreads [150]. The resistome encompasses the complete set of genes and genetic elements within a pathogen that confer resistance to antimicrobial agents. Profiling the resistome can be achieved using various sequencing technologies, including Oxford Nanopore’s MinION for real-time, long-read sequencing, as well as Illumina and Ion Torrent platforms for high-throughput, short-read genomic analysis. Furthermore, host transcriptomics is a powerful approach to identify RNA biomarkers that can aid in the early diagnosis of sepsis, patient stratification, prognosis, and therapeutic monitoring. Direct RNA sequencing with Oxford Nanopore on sepsis patient blood revealed full-length isoform profiles, poly(A) tail length variations, while it identified novel transcriptomic biomarkers differentiating bacterial from viral sepsis [151]. Technologies used include RNA-Seq (Illumina, Ion Torrent), and Nanopore for long-read sequencing of transcriptomes [152]. The host response is a critical aspect of infection and encompasses the immune response, including inflammatory pathways (e.g., NF-κB, JAK-STAT, MAPK signaling), immune cell activation, and cytokine storms. Host responses can be studied at the transcriptomic level or through other omics approaches such as proteomics, metabolomics, and immunomics. Understanding how the host’s immune system is overactive (e.g., in sepsis) or underactive (e.g., in immunocompromised patients) can provide valuable therapeutic insights [153]. The integration and interpretation of multi-omic data, encompassing the resistome, transcriptome, and host response, presents several challenges due to the high dimensionality, heterogeneity, and complexity of the data. However, the development of standardized protocols for data collection, integration, and interpretation is necessary [154].

### 7.4. Global Standards for Resistome Reporting in BSIs

Resistome reporting for BSIs is a critical component in the diagnosis, treatment, and management of infections, particularly in hospitalized patients. As AMR continues to rise globally, the timely identification and reporting of resistance genes in bloodstream infections are vital for personalized medicine and antibiotic stewardship. The need for global standards in resistome reporting has been emphasized to ensure the consistency, reproducibility, and interoperability of AMR data across different health systems. WGS is becoming the gold standard for comprehensive resistome profiling as it provides the most accurate and detailed information on the genetic basis of resistance. Targeted sequencing panels for common resistance genes (e.g., blaTEM, mecA, gyrA) can be used in clinical settings for faster results but may not capture all resistance determinants [73].

Resistome data should be integrated with phenotypic susceptibility data. For example, genomic data from WGS can identify resistance genes, but phenotypic AST can confirm whether these genes translate to resistance in practice. The integration of genomic, transcriptomic, and proteomic data could provide more comprehensive insight into antimicrobial resistance mechanisms [155]. Data standardization and interoperability are critical to ensure that resistome reports can be shared across institutions and countries. Standardized formats, such as FASTQ or VCF (Variant Call Format) for genomic data, and adoption of frameworks like HL7 or FHIR for clinical data, can enable seamless sharing [156]. Common metadata that describes the organism, resistance profiles, and clinical context must be included in reports to ensure accurate interpretation across settings.

## 8. Conclusions

Sequencing technologies have reshaped the landscape of AMR detection in bloodstream infections, but their clinical value depends on selecting the appropriate approach for the diagnostic question at hand. Based on the evidence synthesized in this review, no single platform is universally optimal, and each technology occupies a distinct role within the clinical workflow. WGS offers the highest genomic resolution and is best suited for strain typing, outbreak investigation, and comprehensive resistome characterization but is not typically used for acute, time-critical diagnostics. tNGS provides a faster and more targeted alternative, making it particularly valuable when rapid detection of predefined pathogens or AMR genes is required, especially in institutions with established molecular workflows. mNGS enables unbiased, culture-independent detection and is uniquely advantageous in culture-negative, polymicrobial, or diagnostically ambiguous BSIs, although challenges such as background host DNA, cost, and variable sensitivity still limit its routine use. Long-read sequencing technologies show considerable promise for resolving complex genomic structures and plasmid-mediated resistance but remain emerging tools with limited widespread clinical implementation.

The key message is that sequencing should be deployed strategically, with the choice of platform guided by clinical urgency, laboratory infrastructure, and the specific informational needs of each case. As benchmarking studies expand, protocols become standardized, and analytical pipelines mature, sequencing-based diagnostics are expected to transition from specialized applications to a more integrated role in routine patient care. Ultimately, the thoughtful and evidence-based use of these technologies will enable earlier, more accurate, and more tailored antimicrobial therapy, strengthening clinical outcomes while supporting global efforts to mitigate antimicrobial resistance.

## Figures and Tables

**Table 1:** Comparative performance characteristics of major sequencing platforms used in the detection of antimicrobial resistance in bloodstream infections.

Feature	Illumina (e.g., MiSeq/NextSeq)	Ion Torrent (e.g., S5/Ion Proton)	Oxford Nanopore (e.g., MinION)	PacBio (e.g., Sequel IIe, HiFi)
**Read Type**	Shortread	Shortread	Longread	Longread (HiFi)
**Read Length**	75–300 bp	200–600 bp	10–100 kb (up to Mb)	10–25 kb (HiFi reads)
**Accuracy (Raw Reads)**	>99.9%	~98–99%	~90–95% (improving)	>99.9% (HiFi)
**Diagnostic yield**	~0.3–15 Gb per MiSeq run	~0.3–15 Gb per Ion S5 run	~2–20 Gb per MinION run	~30 Gb per Sequel IIe run
**Turnaround Time**	~24–48 h	~12–24 h	Real-time (~minutes–hours)	~24–48 h
**Library Prep Time**	4–6 h	2–4 h	1–2 h	4–8 h
**Cost per Gb**	~USD 31/Gb for some kits (e.g., 600-cycle)	Variable (~USD 30–300/Gb depending on chip and run)	Variable (~USD 11–33/Gb)	High (but decreasing) ~USD 31–43/Gb for HiFi
**Instrument Cost**	~USD 99,000 for MiSeq/~ USD 210,000 for NextSeq 1000	USD 75,500 for the S5 system	~USD2999–4950 for the device	~USD 495,000 for the Sequel II system
**Strengths**	High accuracy, established pipelines	Fast prep, scalable, affordable runs	Portability, longreads, real-time	High accuracy long reads (HiFi)
**Limitations**	Limited for large repeats or SVs	Lower accuracy than Illumina	Higher error rate, data variability	Higher cost, longer prep

SVs = Structural Variants, Gb = Gigabases, Mb = Megabases.

**Table 2:** Summary of clinical studies evaluating the impact of NGS on patient management and outcomes in ICU and sepsis cohorts.

Study	Cohort	Clinical Impact	Patient-Level Outcomes
Chen et al. [129]	130 sepsis patients (65 mNGS vs. 65 matched control)	Antibiotic regimen was changed in 72.3% in mNGS patients vs. 53.9% in control	Lower mortality in patients who had mNGS early (<24 h) vs. prolonged antibiotic exposure (22.2% vs. 42.9%)
Qin et al. [130]	194 patients (112 with mNGS, 82 without)	Faster pathogen detection (mean 1.41 days via mNGS vs. 4.82 days via conventional methods)	28-day mortality: 47.3% in mNGS group vs. 62.2% in non-mNGS group (*p* = 0.043)
Zuo et al. [131]	277 patients	mNGS sensitivity: 90.5% vs. 36.0% for blood culture; mNGS guided antibiotic modification	30-day survival data; higher pathogen reads by mNGS correlated with mortality risk
Pan et al. [132]	69 sepsis patients	mNGS on blood + infection sites increased pathogen detection compared to conventional methods	Demonstrated that multi-site mNGS can inform more precise treatment decisions in ICU sepsis patients
Li et al. [133]	308 sepsis patients (92 immunocompromised)	mNGS sensitivity much higher than culture (88.0% vs. 26.3% overall), prompting antibiotic changes in 60.1% of cases	Clinical benefit in 76.3% of patients
Chen et al. [134]	97 candidemia patients (blood mNGS)	mNGS revealed microbial co-detections not seen by conventional diagnostics	28-day mortality 44.3%; distinct microbial patterns in non-survivors
